# Misaligned Attitudes and Perceptions Among Adolescents Living With Obesity, Caregivers and Healthcare Professionals: ACTION Teens Australia Survey Study

**DOI:** 10.1111/jpc.70146

**Published:** 2025-07-14

**Authors:** Cathy Kwok, Nicholas Bentley, Jacqueline Curran, Natalie Lister, Helen Truby, Louise A. Baur

**Affiliations:** ^1^ Children's Hospital Westmead Clinical School, The University of Sydney Sydney Australia; ^2^ Novo Nordisk A/S Sydney Australia; ^3^ Perth Children's Hospital Nedlands Australia; ^4^ Institute of Endocrinology and Diabetes, The Children's Hospital at Westmead Sydney Australia; ^5^ University of Queensland Brisbane Australia; ^6^ Weight Management Services, The Children's Hospital at Westmead Sydney Australia

**Keywords:** adolescent, body mass index, clinician, obesity, survey

## Abstract

**Aims:**

To explore perceptions, attitudes, behaviours and barriers relating to adolescent weight management in Australia.

**Methods:**

ACTION Teens was a cross‐sectional, survey‐based study. Adolescents with high body mass index (BMI), caregivers and healthcare professionals (HCPs) from 10 countries completed an online survey in 2021. This analysis was limited to participants in Australia. Adolescents (*N* = 298) were aged 12–< 18 years with BMI ≥ 95th percentile for age and sex. Caregivers (*N* = 276) lived with an eligible adolescent and were involved in healthcare decisions. HCPs (*N* = 137) had ≥ 2 years' clinical experience and saw/treated ≥ 10 adolescents with high BMI per month. Outcomes included perceptions of high BMI, weight loss (history, barriers, definition of success), weight‐management information sources, and history/assessment of weight‐related conversations.

**Results:**

Most adolescents believed their health was good/very good/excellent (83%) but worried about weight impacting their future health (69%). More caregivers indicated their adolescent's health was good/very good/excellent (92%). More adolescents than caregivers agreed weight loss was entirely the adolescent's responsibility (72% vs. 28%), reported a recent weight‐loss attempt by the adolescent (52% vs. 21%) and believed initiating weight‐related discussions with HCPs was the adolescent's responsibility (62% vs. 51%). Only 42% of adolescents had recently discussed weight with an HCP; although 66% of this subset trusted their HCP's advice, the adolescents reported both positive (73%) and negative (44%) feelings following discussions.

**Conclusions:**

To improve adolescent obesity care in Australia, improved communication between adolescents and caregivers/HCPs is needed. We recommend HCPs raise the topic of weight with adolescents in a sensitive manner.

ClinicalTrials.gov identifier: NCT05013359.

**Summary**

- What is already known on the topic?
  ○ In Australia, few adolescents presenting to primary care for weight management receive appropriate assistance.
  ○ Previous studies have highlighted barriers for healthcare professionals working in weight management.
- What this paper adds?
  ○ Through surveying adolescents with a high body mass index, caregivers and healthcare professionals in Australia, we identified misaligned perceptions and attitudes towards weight that may contribute to caregivers underestimating the impact of high body mass index on adolescents' health.
  ○ More adolescents than caregivers and healthcare professionals reported that adolescents should be responsible for weight management and raising weight‐related conversations.
  ○ Healthcare professionals need to be cognisant of stigmatising language during consultations and consider the adolescent's feelings and health concerns.

## 1 Introduction

Many children and adolescents in Australia live with overweight or obesity, with an estimated prevalence of 27.7% in 2022 [1]. Despite adolescents with a high body mass index (BMI) having increased risk of cardiometabolic and psychosocial comorbidities [2, 3], few adolescents with a high BMI in Australia receive appropriate weight‐management assistance from healthcare professionals (HCPs) in the primary‐care setting [4, 5]. This is potentially due to limited time [6, 7], lack of training/confidence [7, 8], limited availability of (or access to) multidisciplinary paediatric weight‐management services [9] and/or stigmatisation of people with a high BMI [10].

While we have some understanding of the barriers experienced by HCPs [6, 7, 8], our understanding of the experiences, challenges and needs of adolescents with a high BMI in Australia is poor. There is limited evidence documenting adolescents' lived experiences and exploring whether caregiver/HCP perceptions differ on this.

In the multinational, cross‐sectional ACTION Teens study, adolescents with a high BMI, their caregivers and their HCPs completed a survey exploring perceptions, attitudes and behaviours relating to weight and weight management [11]. We present ACTION Teens data from Australia and discuss local barriers to the effective management of high BMI among adolescents.

## 2 Materials and Methods

### 2.1 Study Design and Objective

The primary objective of the global, cross‐sectional ACTION Teens survey study (ClinicalTrials.gov, NCT05013359) was to identify perceptions, attitudes, behaviours and potential barriers relating to effective weight management among adolescents with a high BMI, their caregivers and their HCPs.

The study adhered to the principles of the Declaration of Helsinki, the EPHMRA Code of Conduct and all applicable data‐management regulations. The full methods for the global study have been reported previously [11].

### 2.2 Survey Development and Rollout

The ACTION Teens steering committee assisted with the development and approval of survey materials, including the overlapping questionnaires for each respondent group [11]. The participant invitations and complete survey text have been reported previously [11]. The primary outcome measures, which have also been reported for the global study [11], were assessed using single‐ or multiple‐item selection from a defined list (including yes/no responses), Likert scales, or numeric entries. Primary outcomes included perceptions of high BMI and its impact, weight loss (history, barriers, definition of success), weight‐management information sources, and history and assessment of weight‐related conversations between adolescents/caregivers and HCPs.

KJT Group programmed the surveys and collected responses online using Decipher (Forsta, Stamford, Connecticut, USA).

In Australia, participants completed the quantitative online survey (provided in Australian English) between September 21, 2021 and December 13, 2021.

### 2.3 Participants

Only participants who lived/practised in Australia were included in this analysis. Respondents who were age 12–< 18 years and whose self‐reported data indicated a BMI ≥ 95th percentile for those of the same age and sex (according to World Health Organisation charts [12]) were eligible for inclusion in the ‘adolescents with a high BMI’ group (‘adolescents’ hereafter). To be eligible for the caregivers group, respondents (age ≥ 25 years) were required to reside with an eligible adolescent ≥ 50% of the time and participate in decision‐making related to their adolescent's healthcare. Adolescents (and caregivers) were not eligible if they indicated they/their adolescent were ‘extremely muscular’ or had recently experienced significant changes in weight because of major illness/injury. Finally, HCP respondents were required to have ≥ 2 years' clinical practice experience, directly care for patients ≥ 50% of the time and see/treat ≥ 10 adolescents with high BMI in an average month. Electronic informed consent was collected from all respondents (plus each adolescent's parent/legal guardian) before study participation.

Adolescents and caregivers were recruited from online, general‐population consumer panels/databases. HCPs were recruited from online physician panels/databases. An adult general‐population sample (stratified per demographic targets for the Australian general population [region, age, sex, race/ethnicity, education, income]) was targeted and screened to identify eligible caregivers. All eligible caregivers were invited to complete the caregiver survey and asked to consent to their adolescent's completion of the adolescent survey. This enabled recruitment of adolescents through their caregivers, maximising recruitment of ‘matched pairs’. Non‐matched adolescents and caregivers were also recruited to increase participant numbers in pursuit of the desired sample size.

The target sample size was 450 adolescents, 450 caregivers and 150 HCPs in Australia. This sample size considered the need for sufficient statistical power while ensuring recruitment feasibility.

### 2.4 Data Analysis

KJT Group used Excel (Microsoft, Redmond, Washington, USA), SPSS Statistics 23.0 (IBM, Armonk, New York, USA) and Stata IC 14.2 (StataCorp LLC, College Station, Texas, USA) to analyse deidentified data and generate descriptive statistics for all completed surveys. KJT Group removed outliers and reduced base sizes accordingly where appropriate (e.g., for continuous variables).

Demographic weighting of caregiver data (using representative Australian targets for region, age, sex, education and household income) was performed to improve the generalisability of the findings. As such, when reporting proportions of caregivers, the data are weighted; however, absolute numbers are provided when reporting caregiver participant numbers. Adolescent data were not weighted because publicly available demographic data for adolescents were not available for all countries that participated in ACTION Teens. Weights applied to caregiver data could not be applied to adolescent data because not all adolescent and caregiver respondents were matched pairs.

## 3 Results

### 3.1 Participant Characteristics

This analysis used data collected from 298 adolescents, 276 caregivers and 137 HCPs in Australia (Figure S1). Participant demographics and characteristics are shown in Table 1. In total, 11% of adolescents (*n* = 33/298) and 12% of caregivers (*n* = 33/276) were part of matched pairs. Of note, HCPs were primary‐care physicians (74% [*n* = 101/137]), general paediatricians (18% [*n* = 25/137]) or paediatric endocrinologists (8% [*n* = 11/137]), with a mean of 19.7 (standard deviation: 8.7) years in practice.

**TABLE 1:** Participant demographics/characteristics.

	Adolescents	Caregivers	HCPs
Full Australia sample: *N*	298	276	137
Matched pair (adolescent and caregiver): *n* (%)	33 (11)	33 (12)	N/A
Unmatched, *n* (%)	265 (89)	243 (88)	N/A
Mean age: years (SD)	14.8 (1.6)	42.6 (9.7)	51.5 (10.4)
Male: *n* (%)^a^	178 (60)	109 (39)	94 (69)
Female: *n* (%)^a^	120 (40)	167 (61)	43 (31)
Obesity class of adolescent participants, the adolescents of the caregiver participants and the adolescents treated by the HCP participants^b^			
Obesity Class I	53 (*n* = 157)	51 (*n* = 140)	67 (SD: 21)
Obesity Class II	19 (*n* = 56)	21 (*n* = 59)	22 (SD: 14)
Obesity Class III	29 (*n* = 85)	28 (*n* = 77)	11 (SD: 11)
BMI classification of caregiver and HCP participants: *n* (%)^c^			
Underweight (< 18.5 kg/m^2^)	N/A	15 (5)	4 (4)
Normal weight (18.5–24.9 kg/m^2^)	N/A	85 (31)	60 (55)
Overweight (25.0–29.9 kg/m^2^)	N/A	65 (24)	38 (35)
Obesity Class I–III (≥ 30.0 kg/m^2^)	N/A	111 (40)	7 (6)

Abbreviations: BMI, body mass index; HCP, healthcare professional; N/A, not applicable; SD, standard deviation.

^a^ Proportions of male and female participants are based on responses to Q5 in the adolescent/caregiver survey (‘were you born a male or female?’) and Q905 in the HCP survey (‘are you male, female, or other?’).

^b^ Obesity Class I indicates a BMI ≥ 95th percentile for age and sex; Obesity Class II indicates a BMI ≥ 120% of 95th percentile for age and sex; and Obesity Class III indicates a BMI ≥ 140% of 95th percentile for age and sex. For adolescents and caregivers, data show the percentage (number) of adolescents; for HCPs, data show the mean percentage (SD) of their adolescent patients.

^c^ *n* = 109 for HCP BMI classification. Caregiver demographic data are unweighted. Table adapted from [11]. BMI, body mass index; HCP, healthcare professional; N/A, not applicable; SD, standard deviation.

### 3.2 Perceptions of High BMI and its Impact

**FIGURE 1:**
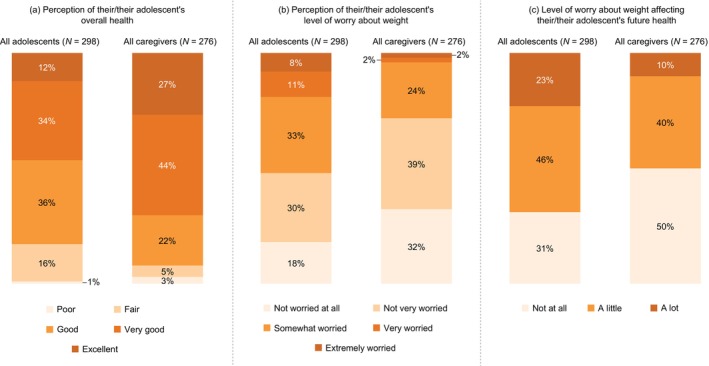
Adolescent and caregiver (a) perception of their/their adolescent's overall health, (b) perception of their/their adolescent's level of worry about weight, and (c) level of worry about weight affecting their/their adolescent's future health. Data show the proportion of adolescents and caregivers who selected each prespecified response option. Percentages may not sum to 100% due to rounding. Figure adapted from [11].

Fewer adolescents than caregivers indicated their/their adolescent's health was at least good (83% [*n* = 247/298] vs. 92% [*n* = 256/276]) and at least very good (47% [*n* = 139/298] vs. 70% [*n* = 186/276]). Additionally, most adolescents (52% [*n* = 154/298]) reported being at least somewhat worried about their weight; by comparison, only 29% of caregivers (*n* = 98/276) thought their adolescent was at least somewhat worried (Figure 1). Similarly, a greater proportion of adolescents (69% [*n* = 205/298]) than caregivers (50% [*n* = 154/276]) indicated that they worry at least a little about weight affecting their/their adolescent's future health (Figure 1). While 42% of caregivers (*n* = 118/276) thought that their adolescent's weight made their adolescent unhappy sometimes/often/always, most adolescents (60% [*n* = 180/298]) indicated this.

### 3.3 Weight Loss

**FIGURE 2:**
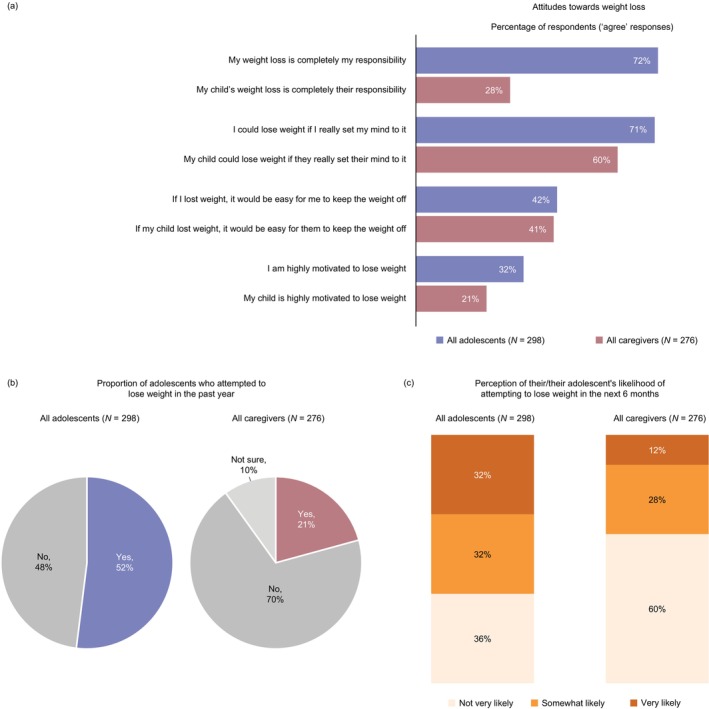
Adolescent and caregiver (a) attitudes towards weight loss, (b) perception of their/their adolescent's recent weight‐loss attempts and (c) perception of their/their adolescent's likelihood of attempting to lose weight in the next 6 months. Data show the proportion of adolescents and caregivers with relevant response options. In panel (a), the proportion of participants who ‘agree’ is the sum of those who selected ‘strongly agree’ or ‘somewhat agree’. In panel (c), the proportion of participants in the ‘not very likely’ category is the sum of those who selected ‘not likely at all’ or ‘not very likely’, while the proportion in the ‘very likely’ category is the sum of those who selected ‘very likely’ and ‘extremely likely’. Percentages may not sum to 100% due to rounding. Figure adapted from [11].

More adolescents than caregivers agreed that the adolescent was completely responsible for losing weight (72% [*n* = 214/298] vs. 28% [*n* = 80/276]), reported that the adolescent had made a weight‐loss attempt within the past year (52% [*n* = 156/298] vs. 21% [*n* = 64/276]), and reported that the adolescent was likely to make a weight‐loss attempt over the next 6 months (64% [*n* = 190/298] vs. 40% [*n* = 115/276]) (Figure 2).

Adolescent and caregiver responses indicated that the weight‐management methods most used by adolescents in the past year were improving their eating habits (44% of adolescents [*n* = 131/298] and caregivers [*n* = 119/276]) and becoming more physically active (38% of adolescents [*n* = 113/298] and caregivers [*n* = 107/276]), although 24% of adolescents (*n* = 72/298) had not used any weight‐management methods in the past year.

Adolescent responses suggested that their top weight‐loss barriers were lack of motivation (37% [*n* = 111/298]), enjoyment of unhealthy food (30% [*n* = 89/298]) and an inability to control their hunger (27% [*n* = 79/298]). However, some adolescents indicated that there were no barriers preventing them from losing weight (23% [*n* = 68/298]).

Adolescents most often defined successful weight loss as generally feeling healthier (48% [*n* = 142/298]) and feeling better about themselves (45% [*n* = 133/298]), with approximately one‐third (34% [*n* = 102/298]) defining successful weight loss as having improved mental health (i.e., less depression or anxiety).

### 3.4 Weight‐Management Information Sources

Although 22% of adolescents (*n* = 66/298) indicated they had not used any sources of information to learn about weight management, use of internet‐based resources including YouTube (35% [*n* = 104/298]), social media (28% [*n* = 84/298]) and search engines (27% [*n* = 80/298]) was common. By comparison, only 15% of adolescents (*n* = 46/298) reported using information from a doctor, although a greater proportion of caregivers (27% [*n* = 82/276]) reported they had used information from a doctor.

### 3.5 Weight‐Related Conversations

Only 42% of adolescents (*n* = 126/298) had discussed their weight with an HCP in the past year; among this subset (excluding eight outliers), 40% (*n* = 47/118) had discussed weight with an HCP once, whereas 22% (*n* = 26/118) had discussed it ≥ 5 times. By comparison, 40% (111/276) caregivers had discussed their adolescent's weight with an HCP in the past year; among this subset (excluding one outlier), only 5% (*n* = 10/110) reported that their adolescent had discussed weight with an HCP ≥ 5 times.

**FIGURE 3:**
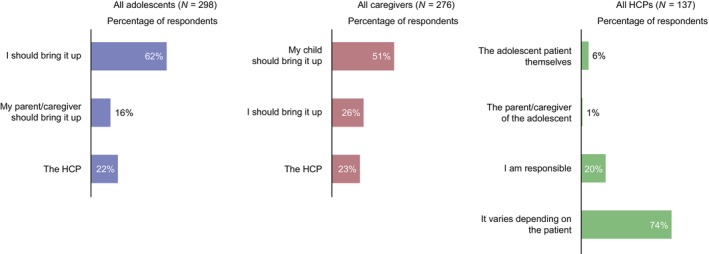
Adolescent, caregiver and HCP perceptions of who is responsible for initiating weight‐related discussions during HCP appointments. Data show the proportion of adolescents, caregivers and HCPs who selected each prespecified response option. Figure adapted from [11].

Among the subsets of adolescents and caregivers who had discussed their/their adolescent's weight with an HCP, 43% (*n* = 54/126) and 30% (*n* = 33/111), respectively, indicated the adolescent was usually the first to raise the topic. However, HCPs reported that adolescent patients raise the topic of weight 19% of the time on average (standard deviation: 16.8). Only 6% of HCPs (*n* = 8/137) thought it was the adolescent's responsibility to initiate weight‐related discussions, whereas 62% of adolescents (*n* = 184/298) and 51% of caregivers (*n* = 126/276) believed it was the adolescent's responsibility (Figure 3).

Approximately one‐fifth of adolescents (21% [*n* = 64/298]) reported that already knowing what they need to do to manage their weight is a barrier to discussing it with an HCP, although 29% of adolescents (*n* = 85/298) indicated there is nothing preventing such discussions.

**FIGURE 4:**
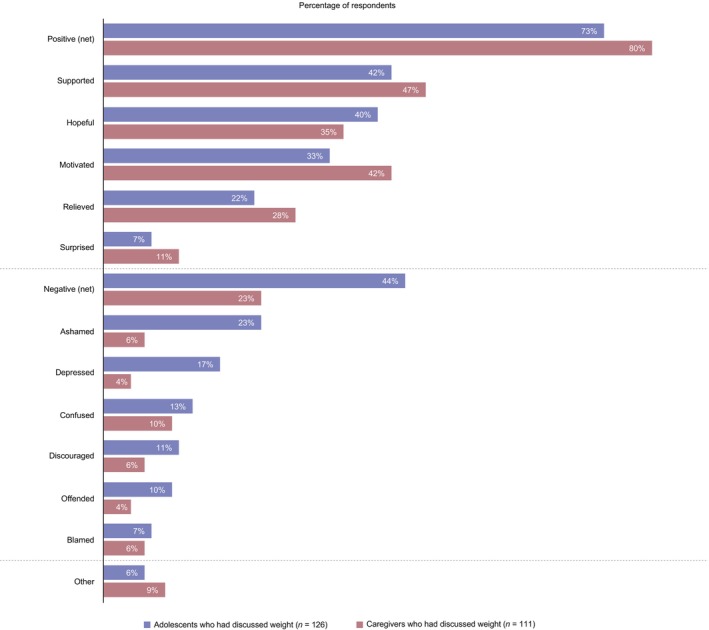
Adolescent and caregiver feelings after most recently discussing weight with an HCP. Data show the proportion of adolescents and caregivers who had discussed weight with their/their adolescent's HCP in the past year and selected each prespecified response option in relation to their own feelings after the discussion. The net‐positive category includes all participants who selected ≥ 1 positive answer (i.e., supported, hopeful, motivated, relieved and/or surprised); the net‐negative category includes all participants who selected ≥ 1 negative answer (i.e., ashamed, depressed, confused, discouraged, offended and/or blamed). As this was a quantitative study, there are no qualitative data available for respondents who selected the ‘other’ response option. Figure adapted from [11].

Adolescents and caregivers who had discussed their/their adolescent's weight with an HCP reported both positive and negative feelings after their most recent weight‐related discussion (Figure 4). Approximately three‐quarters of adolescents (73% [*n* = 92/126]) reported at least one positive feeling, most often feeling supported (42% [*n* = 53/126]), hopeful (40% [*n* = 50/126]) and motivated (33% [*n* = 41/126]); similarly, many caregivers (80% [*n* = 95/111]) reported at least one positive feeling. However, 44% of adolescents (*n* = 55/126) reported at least one negative feeling, most commonly feeling ashamed (23% [*n* = 29/126]) and depressed (17% [*n* = 21/126]); a lower proportion of caregivers reported experiencing negative feelings (23% [*n* = 26/111]). Despite this, most adolescents who had discussed weight with an HCP agreed they trust their HCP's weight‐management advice (66% [*n* = 83/126]).

## 4 Discussion

In this sub‐analysis of ACTION Teens data, survey responses highlight discrepancies between adolescents with a high BMI, caregivers, and HCPs in relation to perceptions of health and adolescents' responsibility in weight management and weight‐related conversations.

Of note, a greater proportion of adolescents than caregivers reported a recent weight‐loss attempt by the adolescent and felt weight loss was entirely the adolescent's responsibility. Additionally, most adolescents indicated that their health was at least good, despite a high proportion worrying about weight affecting their future health. However, a greater proportion of caregivers than adolescents believed the adolescent's current health was at least good, and fewer caregivers were worried about weight affecting their adolescent's future health. This suggests a lack of communication between adolescents and their caregivers regarding the impact of living with a high BMI on health and psychological wellbeing. This finding is consistent to previous Australian survey studies [13, 14] and a meta‐analysis of international studies [15] suggesting caregivers often underestimate the weight status of their adolescent with a high BMI. Interestingly, a previous qualitative study in Australia identified a lack of parental recognition of their adolescent's excess weight as a factor that may reduce overall receptivity and adherence to community weight‐management interventions [16]. Caregivers with a high BMI are also more likely to perceive their overweight children as having normal weight [17]. Notably, parents with a high BMI are also likely to have children with a high BMI [18]. Taken together, these findings suggest that misaligned perceptions of/attitudes towards weight management between adolescents and caregivers, and poor communication regarding the adolescent's weight status and health, could be important barriers to effective weight management for adolescents.

Previous studies have reported that Australian adolescents with a high BMI are often not appropriately managed by HCPs [4, 5], with HCPs missing opportunities to manage weight during primary‐care consultations [4]. Similarly, we found that under half the surveyed adolescents had recently discussed their weight with an HCP. This may be related to the finding that most adolescents assumed personal responsibility for initiating conversations about weight during HCP appointments, although notably, a lower proportion of caregivers placed responsibility on the adolescent and very few HCPs considered adolescents to be solely responsible. Broadly, we also recognise there are several access issues that can prevent adolescents from engaging with HCPs, such as the need for a parent to be present and/or to take them to appointments, along with a lack of culturally appropriate services [19, 20].

Despite weight‐management advice being generally well received by adolescents who discuss weight with their HCP, the negative feelings experienced after recent discussions about weight highlight the importance of HCPs raising the topic sensitively. This requires HCPs to have self‐awareness of their own weight bias and attitudes regarding adolescents with a high BMI [21, 22]. The finding that adolescents appear to assume personal responsibility for weight management is something HCPs should be aware of, as high BMI is associated with impaired psychosocial health [23], and adolescents with a high BMI are more likely to engage in self‐directed unhealthy and extreme weight‐control behaviours [24, 25]. Adolescents with a high BMI should be made aware of the biological basis of excess weight, and the challenges impeding weight management. HCPs should highlight long‐term weight‐management goals that focus on the psychosocial aspects commonly identified by adolescents as markers of success, i.e., generally feeling healthier, feeling better about themselves and improved mental health. This approach is likely to improve overall communication between adolescents and HCPs in Australia and could potentially reduce the likelihood of adolescents assuming excessive personal responsibility for their weight.

### 4.1 Limitations

The cross‐sectional design of ACTION Teens precludes identification of causal relationships and analysis of long‐term trends. Additionally, it is possible that the use of self‐reported survey data may have led to under‐ or over‐estimation of adolescents' BMI and thus affected adolescent and caregiver eligibility for the study. While sex‐based analyses fall outside of the scope of this paper, it should be noted that only 40% of the adolescents surveyed in Australia (*n* = 120/298) identified as female; as such, our findings may be biased towards the perceptions/experiences of male adolescents. Despite this, we believe our findings are generally representative of the wider population of adolescents with a high BMI (and their caregivers) in Australia, as ACTION Teens was designed to reduce the risk of sampling bias by, for example, stratifying the general‐population sample used for recruitment of adolescents and caregivers and weighting caregiver data to representative local demographic targets. Another limitation is that only around 10% of adolescents and caregivers were recruited as ‘matched pairs’; therefore, differences in perceptions among the adolescent and caregiver groups may not be reflective of differences between adolescent–caregiver pairs. Furthermore, the sample size was lower in Australia than originally planned, as the recruitment period was shorter than anticipated, and fielding was delayed while awaiting ethical approval. Finally, it would be beneficial to conduct qualitative interviews with adolescents who have a high BMI, their caregivers and their HCPs to enhance our understanding of their lived experiences and barriers to effective weight management.

## 5 Conclusion

Results from ACTION Teens suggest that just over half of Australian adolescents with a high BMI are worried about their weight and have recently tried to lose weight. However, some perceptions and attitudes appear to be misaligned among adolescents and caregivers. More adolescents than caregivers were concerned about the impact of a high BMI on adolescents' future health and held adolescents personally responsible for losing weight and initiating weight‐related conversations with HCPs, and the findings highlight a need for improved communication between adolescents with a high BMI and their caregivers in Australia. Additionally, HCPs are recommended to provide non‐stigmatising care that considers adolescents' feelings and health concerns.

## Author Contributions

L.A.B contributed to the design of the study. All authors participated in data interpretation and drafting/revising the manuscript and approved the final, submitted version of the manuscript.

## Ethics Statement

The ACTION Teens study for Australia was approved by the University of Sydney Human Research Ethics Committee on September 20, 2021 (reference number: 2021/634).

## Conflicts of Interest

C.K. reports support from Novo Nordisk to attend and present this research at the Australian and New Zealand Obesity Society Annual Scientific Meeting 2023. N.B. is an employee of Novo Nordisk. J.C. is site Principal Investigator for the PIONEER TEENS clinical trial, which is funded by Novo Nordisk. N.L. and H.T. report no competing interests. L.A.B. reports consultancy fees from Novo Nordisk during the conduct of the study (for her role as a member of the ACTION Teens steering committee) and speaker fees from Novo Nordisk outside the submitted work.

## Supporting information


**Data S1.** Supporting Information.

## Data Availability

All authors had full access to all of the data related to this analysis. Data will be shared with bona fide researchers submitting a research proposal approved by the independent review board. Individual participant data will be shared in datasets in a deidentified and anonymised format. Data will be made available after research completion. Information about data access request proposals can be found at novonordisk‐trials.com.
