# Supplementary material for: Misaligned Attitudes and Perceptions Among Adolescents Living With Obesity, Caregivers and Healthcare Professionals: ACTION Teens Australia Survey Study
Source: J Paediatr Child Health. 2025 Jul 14;61(10):1573–81. doi: 10.1111/jpc.70146 (PMC12515272; doi:10.1111/jpc.70146)
Supplement: Supplementary file 1 — Data S1. Supporting Information. [file JPC-61-1573-s001.docx]

# Online Supporting Information

**Misaligned Attitudes and Perceptions Among Adolescents Living With Obesity, Caregivers and Healthcare Professionals: ACTION Teens Australia Survey Study**

**Fig. S1** Sample disposition for Australian participants: (a) adolescents and caregivers, and (b) HCPs.

**(a)**

*N* = 22,224

**Responded**

*n* = 4671 (21.0%)

**Screening suspends**

*n* = 17,553 (79.0%)

**Completed screener**

*n* = 16,772 (95.6%)

**Screening exclusions**

*n* = 781 (4.4%)

**Qualified**

*n* = 81 (10.4%)

**Over quota**

*n* = 700 (89.6%)

**Quota qualified**

*n* = 53 (7.6%)

**Post-qualification suspends**

*n* = 647 (92.4%)

**Completed body**

*n* = 73 (11.3%)

**Data validation fail**

*n* = 574 (88.7%)

**Valid complete**

*n* = 298

**Adolescents**

*n* = 276

**Caregivers**

**Exclusion reasons**

Does not agree to consent

Does not agree to adverse event

Caregiver age 18–24 years or child age < 12 years

Is not the primary caregiver of an adolescent

Birth date unknown by respondent or no adolescent age 12–< 18 years

Does not reside in a participating country

Declined to answer income

Declined to answer ethnicity

None of the children in the household are age 12–< 18 years

No qualified adolescent in the household

No adolescents age 12–< 18 years with high BMI in the household

Adolescent had significant weight loss/gain in the past 6 months

Adolescent is extremely muscular

**(b)**

*n* = 537 (5.7%)

**Responded**

*n* = 60 (11.2%)

**Screening suspends**

*n* = 477 (88.8%)

**Completed screener**

*n* = 330 (69.2%)

**Screening exclusions**

*n* = 147 (30.8%)

**Qualified**

*n* = 0 (0%)

**Over quota**

*n* = 147 (100.0%)

**Quota qualified**

*n* = 9 (6.1%)

**Post-qualification suspends**

*n* = 138 (93.9%)

**Completed body**

*n* = 1 (0.7%)

**Data validation fail**

**Exclusion reasons**

Does not agree to consent

Does not agree to adverse event

Does not practise in a participating country

Not a physician

Not a qualified speciality

Less than 50% of time spent in patient care

Less than 2 years in practice

Has not seen minimum required patients

Has not seen required number of adolescent patients with high BMI per month

*n* = 137 (99.3%)

**Valid complete**

*N* = 9418

**Survey invitations sent**

*n* = 8881 (94.3%)

**No response**

Screening suspends (i.e., screening dropout): the qualification section of the survey was not completed. Over quota: respondent excluded as the target for completed surveys matching the respondent’s qualification criteria had already been met. Post-qualification suspends (i.e., survey dropout): the main body of the survey was not fully completed. Data validation fail: respondent excluded from the final dataset on the basis of failed data validation checks (e.g., evidence of ‘straight-lining’ on Likert scales, inaccurate responses to data validity questions or very short survey completion time). Figure adapted from J. C. G. Halford, A. Bereket, B. Bin-Abbas, et al., “Misalignment Among Adolescents Living With Obesity, Caregivers, and Healthcare Professionals: ACTION Teens Global Survey Study,” *Pediatric Obesity* 17, no. 11 (2022): e12957. BMI, body mass index; HCP, healthcare professional.
